# Editorial of Special Issue “Oral Cancer: From Pathophysiology to Novel Therapeutic Approaches”

**DOI:** 10.3390/biomedicines11102748

**Published:** 2023-10-11

**Authors:** Vui King Vincent-Chong

**Affiliations:** Department of Oral Oncology, Roswell Park Comprehensive Cancer Center, Buffalo, NY 14263, USA; vincentvuiking.chong@roswellpark.org; Tel.: +1-7164803829

## 1. Introduction

Oral squamous cell carcinoma (OSCC) is a heterogeneous type of malignancy that develops within the oral cavity comprising the lips, tongue, mouth floor, gums, and buccal mucosa, with more than 90% arising from the oral lining epithelium [1]. Tobacco smoking and excessive alcohol drinking are the major risk factors contributing to OSCC [2]. More recently, HPV virus infections have been documented as another of the risk factors [3]. In 2020, approximately 377,000 new cases and 177,700 OSCC deaths were reported according to Global Cancer Observatory (GLOBOCAN) [4]. According to National Comprehensive Cancer Network (NCCN) guidelines, the major treatment for OSCC is surgery, followed by radiation or chemotherapy or both, which is known as chemoradiation (CRT), depending on the clinical stage [5]. Despite advancements in multimodality treatments for OSCC, the clinical outcomes have remained poor over the past decades [6,7,8,9], with the main reason being the acquirement of resistance to chemo or radiation therapy [10,11]. Accumulating evidence indicates that the failure of cancer treatment or its recurrence may be caused by the cancer cells involved in the mechanisms related to DNA damage repair, cell cycle dysregulation, cancer stem cells that promote self-renewal ability, and epithelial–mesenchymal transition (EMT) [11,12]. This has prompted us to seek new technology and efforts to decipher the pathophysiology of oral cancer and improve the often-ineffective current treatment options. To overcome these challenges, the investigation of novel therapeutic approaches related to immunotherapy, targeted therapy, and precision medicine show much promise in enhancing tumor destruction in the OSCC management process and provide new insights into strategies for improving cancer treatment.

## 2. Pathophysiology of Oral Cancer

OSCC is a malignancy that arises from a multistep molecular mechanism derived from normal epithelium to dysplasia that eventually transforms into invasive SCC via multiple genetic and molecular alterations post exposure to chronic stimulation from environmental factors such as tobacco smoking, excessive alcohol drinking, or exposure to HPV [13]. These major risk factors induce mutation and genetic alterations that activate the cancer hallmarks as described in Hanahan and Weinberg, leading to uncontrolled cell proliferation, the evasion of growth suppressors, replicative immortality, angiogenesis, inflammation, resistance to cell death and invasion, and metastasis [14]. Following the completion of The Cancer Genome Atlas (TCGA) on head and neck SCC (HNSCC) which comprises OSCC, integrative exome sequencing data have revealed hotspot mutations which include *TP53*, *FAT1*, *EPHA2*, *NOTCH1*, *CASP8*, and *PIK3CA* [15,16]. In addition, studies have shown that OSCC is driven by essential cancer pathways related to the EGFR/PI3K/AKT/mTOR cascade which play a significant role in the pathogenesis of OSCC [17]. This has led to studies investigating potential molecular targets from these OSCC-related cancer pathways. The EGFR/PI3K/AKT/mTOR cascade is activated by EGF ligands binding to EGFR, leading to PI3K activation. PI3K activation induces Akt phosphorylation, which activates mTORC1 with phosphorylating key components which trigger downstream activities involved in apoptosis, metabolism, cell proliferation, and cell growth. Numerous efforts have been initiated to target EGFR pathways to halt the PI3K/AKT/mTOR cascade through monoclonal antibodies targeting the ligand binding domain of the receptor and the small-molecule tyrosine kinase inhibitors (TKIs) in HNSCC clinical trials [18,19]. However, overall survival rates from the random clinical trial (RCT) studies focusing on recurrent/metastasis (R/M) HNSCC patients remain below 50%. This highlights the importance of identifying biomarkers for predicting the clinical responses of these targeted therapies in HNSCC patients who could benefit from these regimens.

## 3. Treatment Options for Oral Cancer

According to NCCN guidelines, the standard of care for OSCC patients depends on the clinical staging comprising surgery followed by radiation therapy and/or chemotherapy, known as CRT [5]. Patients diagnosed with recurrent/metastasis are treated with immunotherapy as an alternative standard of care. Generally, cisplatin remains the main chemotherapeutic agent for OSCC patients [5]. Despite the potent anti-cancer activities of cisplatin shown in preclinical OSCC models, the development of cisplatin resistance in cancer cells impairs the cytotoxicity effect of this regimen and leads to ineffective treatment as evidenced by a meta-analysis report showing that more than 30% of HNSCC patients acquired cisplatin resistance [10,11]. Over the past decades, there has been growing interest in developing novel therapeutic interventions for OSCC aimed at selectively targeting cancer cells while sparing normal cells to reduce the adverse effects associated with conventional standards of care [20,21,22]. The RCT CHECKMATE 141 studies in R/M HNSCC patients who failed cisplatin treatment report that nivolumab treatment prolongs overall survival rates among such patients compared to conventional standards of care. This has made immunotherapy another promising regimen for such patients despite the response rate being < 20% [9]. Nivolumab, also known as an immune checkpoint blockade (ICB), plays a role in preventing immune evasion by blocking the engagement of PD-L1 in tumors with the PD-1 receptor in CD8 T cells, which could lead to exhaustion in promoting immune surveillance [23]. The below 20% response rate for a single agent of Nivolumab has prompted the emergence of targeted therapies for druggable candidates exposed to therapeutic vulnerabilities in OSCC using personalized medicine based on genomic approaches, transcriptomic profiling, and single-cell sequencing [24]. This facilitates the identification of exclusive druggable hotspot mutations from OSCC patients’ samples and allows them to receive targeted therapies aimed specifically at the genetic mutations or proteins that favor tumor cell growth and metastasis.

## 4. Artificial Intelligence (AI)

Over the past few years, artificial intelligence, an area that involves the capacity of machines to compete with human intellectual capacity, has been widely used in the field of OSCC research to provide more accurate predictions for early diagnosis and prognosis outcomes to improve OSCC management [25]. Generally, AI has two subfields, which are machine and deep learning. Machine learning involves the use of algorithms and computer processes to recognize patterns and provide diagnoses according to input information. Briefly, there are two types of approaches, namely supervised and unsupervised methods. The supervised approach employs a labeled set of training data to map input-to-output data, whereas the unsupervised approach involves analyses of unlabeled datasets using algorithms to discover hidden patterns without human supervision. A systematic review has reported machine learning as a promising emerging approach to accurately classify the differentiation of OSCC and predict prognosis and lymph node metastasis in OSCC patients [26,27]. Deep learning, on the other hand, uses a machine learning method called artificial neural networks to extract patterns and provide predictions from large datasets. Studies have used deep learning approaches in applications for predicting/detecting early diagnosis, metastases and prognosis based on genomic, methylation and transcriptomic data, and pathological and radiographic images of OSCC [28,29]. Overall, deep learning algorithms allow for the identification of biomarkers to predict patient prognosis and guide personalized treatment plans to reduce OSCC burdens and improve the quality of life.

## 5. Limitations, Challenges, and Conclusions

To date, cetuximab and ICB remain the only systemic targeted therapies approved for the treatment of OSCC. However, the clinical benefits of these therapies remain poor with a response rate of less than 20%. This has prompted the search for novel treatment strategies to improve the clinical outcomes for OSCC patients. The lack of significant biomarkers or prognosticators for OSCC remains a challenge in early and advanced stage detection, leading to poor clinical outcomes. Newly emerging interests in genomic, proteomic, transcriptomic, and metabolomic markers for prognostic and predictive markers may facilitate treatment selection. Another challenge for identifying biomarkers in OSCC is the heterogeneity found in tumor characteristics that provide variables of tumor mutational burden. Therefore, non-invasive biomarkers such as tumor oxygenation and signals from autofluorescence imaging, intensity signals from optical coherence tomography, and ultrasound wave signals from surface tumor tissues have been shown to provide accurate predictions for early detection and prognosis assessments in OSCC [30]. To overcome these challenges, this Special Issue has accepted a few publications that highlighted the potential biomarkers that can be used for diagnostic and therapeutic purposes [31,32,33,34,35,36,37,38,39,40]. The diagnostic section can be divided into biomarkers for OSCC and prognosis. In this case, studies have demonstrated that the protein expression of C44Mab-34 [32] can solely detect the OSCC cell as opposed to the stroma or immune cell, and the downregulation of IFIT2 has been identified as a poor prognostic marker for OSCC [33]. A review paper conducted by Yeh et al. [40] summarized evidence showing that TERT promoter mutations were associated with poor survival in OSCC. Apart from the mutations and protein expression of the markers, this Special Issue also provides evidence of using the clinicopathological parameter (lymph node ratio) as a prognosticator to access patient clinical outcome post surgery [36]. Similarly, Chen et al. provided a longitudinal study that demonstrated the significance of using psychological behaviors as a marker to access patients’ requirements for follow-up for appropriate supportive interventions to strengthen patients’ psychosocial adjustments in OSCC patients who had undergone surgery [35]. Another clinical study also demonstrated the potential of facilitating the platelet–lymphocyte ratio (PLR) in OSCC patients prior to surgery treatment as a prognosticator for disease-specific survival (DSS) and progression-free survival (PFS) of patients [38]. This Special Issue also published a comprehensive review that provides an update on recent studies using preclinical models of HNSCC to investigate the role of natural products as therapeutic or chemo preventive agents to improve patient clinical outcomes [39]. In addition to the potential of natural products as therapeutic agents for HNSCC, this Special Issue also published an original article highlighting the role of mometasone furoate (MF) as a potential therapeutic agent targeting PTPN11, which halts tumorigenesis in vitro and impairs tumor growth in vivo using a preclinical model of HNSCC [31]. For the diagnostic approach that focuses on the early detection in OSCC, Surendran et al. [37] demonstrated the gradual increments in the infiltration of Tregs (CD4, CD25, FOXP3), exhausted CD8 T cells (CD8 and PD-1) in the immune microenvironment, and the expression of PD-L1 in epithelial cells in the oral carcinogenesis mechanism, from normal epithelium to dysplasia and eventually to invasive SCC. With the advancement of technology, it is no surprise that our Special Issue also successfully published a paper that focuses on using a deep-learning-based model to automate the identification and visualization of OSCC in optical coherence tomography images from the extracted tumor tissues to classify the normal epithelium, dysplasia, and OSCC based on the features extracted from the OCT images [34]. Using OCT imaging in combination with a deep learning model can act as an adjunct tool for identifying OSCC and margin and sparing the normal region during surgical excision.

In conclusion, OSCC is a deadly malignancy and a major public health issue worldwide. While significant efforts have been made to gain better insights into the pathophysiology of OSCC, current standards of care remain limited and ineffective. Studies that focus on establishing novel therapeutic approaches that involve overcoming chemoresistance and eliminating tumor evasion from immune surveillance are needed to improve the clinical outcomes for OSCC patients.
